# Fibroblast MMP14-Dependent Collagen Processing Is Necessary for Melanoma Growth

**DOI:** 10.3390/cancers13081984

**Published:** 2021-04-20

**Authors:** Elke Pach, Jürgen Brinckmann, Matthias Rübsam, Maike Kümper, Cornelia Mauch, Paola Zigrino

**Affiliations:** 1 Department of Dermatology and Venereology, Faculty of Medicine, University of Cologne, Hospital Cologne, 50937 Cologne, Germany; elke.pach@uk-koeln.de (E.P.); maike.kuemper@uk-koeln.de (M.K.); cornelia.mauch@uk-koeln.de (C.M.); 2 Department of Dermatology, Institute of Virology and Cell Biology, University of Lübeck, 23562 Lübeck, Germany; juergen.brinckmann@uni-luebeck.de; 3 Max Planck Institute for Biology of Ageing, 50931 Cologne, Germany; ruebsam0@uni-koeln.de

**Keywords:** MMP14, proteases, melanoma, collagen

## Abstract

**Simple Summary:**

Matrix metalloproteinases (MMPs) were considered as targets for the treatment of various cancers. However, initial trials using broad inhibitors to MMPs have failed, partly attributed to the contrasting functions of these proteases acting as tumor promoters and suppressors, among other reasons. Our data now suggest that specific inhibition of MMP14 might represent a more specific approach, as loss of this protease in fibroblasts resulted in reduced growth of grafted melanomas. Here, we found that deletion of MMP14 in fibroblasts generates a matrix-rich environment that reduces tumor vascularization and melanoma cell proliferation. In in vitro and ex vivo assays, we showed that the latter is mediated by stiffening of the tissue due to collagen accumulation. Additionally, in vivo, we show that independently of MMP14 deletion, a collagen-rich stiff matrix inhibits the growth of melanomas.

**Abstract:**

Skin homeostasis results from balanced synthesis and degradation of the extracellular matrix in the dermis. Deletion of the proteolytic enzyme MMP14 in dermal fibroblasts (MMP14^Sf−/−^) leads to a fibrotic skin phenotype with the accumulation of collagen type I, resulting from impaired proteolysis. Here, we show that melanoma growth in these mouse fibrotic dermal samples was decreased, paralleled by reduced tumor cell proliferation and vessel density. Using atomic force microscopy, we found increased peritumoral matrix stiffness of early but not late melanomas in the absence of fibroblast-derived MMP14. However, total collagen levels were increased at late melanoma stages in MMP14^Sf−/−^ mice compared to controls. In ex vivo invasion assays, melanoma cells formed smaller tumor islands in MMP14^Sf−/−^ skin, indicating that MMP14-dependent matrix accumulation regulates tumor growth. In line with these data, in vitro melanoma cell growth was inhibited in high collagen 3D spheroids or stiff substrates. Most importantly, in vivo induction of fibrosis using bleomycin reduced melanoma tumor growth. In summary, we show that MMP14 expression in stromal fibroblasts regulates melanoma tumor progression by modifying the peritumoral matrix and point to collagen accumulation as a negative regulator of melanoma.

## 1. Introduction

The tumor microenvironment (TME) consists of numerous different stromal cell types, such as fibroblast and cancer-associated fibroblasts (CAFs), vascular and immune cells, and a scaffold of the extracellular matrix (ECM) [1]. Cross-communication of the cancer cells with the surrounding environment leads to recruitment and reprogramming of stromal cells to promote tumor growth [2].

The non-cellular part of the TME is a dynamic structural network that consists of a variety of extracellular molecules, including collagens, elastin, fibronectin, laminins, and proteoglycans. These proteins are secreted, deposited, and remodeled mostly by fibroblasts, thus ensuring tissue homeostasis or providing a tumor microenvironment supporting tumor growth. Besides its supportive function, the ECM serves as a scaffold for tissue and cells and regulates cellular processes such as proliferation, cell death, migration, or differentiation in physiological and pathological conditions [3]. The composition and the mechanical properties of the TME can promote [4], and in some tumors such as pancreatic cancer [5], restrain cancer progression.

Besides producing ECM proteins, fibroblasts and their activated counterparts, CAFs, regulate the activation of several proteolytic enzymes in healthy skin and tumors. The best characterized in these processes are the matrix metalloproteinases (MMPs) [6,7]. MMP14, a membrane-bound MMP, fulfills an essential role in tissue homeostasis and development. This is evident because its complete deletion in vivo leads to early lethality due to severe organ and bone development defects [8,9,10]. Moreover, by deleting MMP14 in adult mice fibroblasts (MMP14^Sf−/−^), we demonstrated that MMP14 is the major collagenase in skin homeostasis [11]. In different types of cancer, expression and activity of MMP14 were associated with poor prognosis [12]. Additionally, in melanomas, MMP14 is expressed at the leading edge of the invasive front in melanoma and stromal cells in areas of strong MMP2 activation [13]. In both cell types, the matrix is an inducer of expression and activation of MMP2/9 and MMP14 [14,15]. The particular pattern of protease activity correlates with tumor progression [16]. Although MMP14 in melanoma cells has various roles [17], the functional significance of fibroblast MMP14 shaping the tumor microenvironment is less understood. Using MMP14^Sf−/−^ mice, in which the absence of MMP14 in adult fibroblasts leads to enhanced collagen accumulation and tissue tension, we aimed to investigate the specific role of fibroblast MMP14 and the resulting dermal environmental changes in melanoma growth.

## 2. Materials and Methods

### 2.1. Cell Culture

B16F1 GFP cells [18] carry deletions of exons 1α, 1β, and 2 of the Ink4a/Arf gene locus, resulting in loss of p16Ink4a and p19Arf protein expression and altered p53 protein expression without detectable gene mutations, however with no activating BRAF mutations in exons 11 and 15 [19]. B16F1 cells were grown in DMEM medium (+4.5 g/L D-Glucose, L-Glutamine, Thermo Fisher Scientific, Darmstadt, Germany) supplemented with 10% FCS, 2 mM L-glutamine, 100 U/mL penicillin, and 100 µg/mL streptomycin. Cells were counted using a Neubauer cell counting chamber (Laboroptik, Friedrichsdorf, Germany). Cells were routinely tested negative for mycoplasma (PCR Mycoplasma Test Kit I/C; PK-CA91-1096; Promocell, Heidelberg, Germany). For coating, the recombinant proteins (acid extracted Bovine Collagen Type I, Curacyte Discovery GmbH, Leipzig, Germany; human fibronectin, Roche Life Science, Penzberg, Germany) were diluted in PBS and incubated overnight at 4 °C.

### 2.2. Melanoma Spheroids

Spheroids were produced using the liquid overlay method, as previously described [20]. The 5000 B16F1 cells were seeded in a 96-well plate coated with 1.5% agar Noble (BD Biosciences, BD 214220, Heidelberg, Germany) and incubated for 72 h, in which cells organized into a three-dimensional structure. Spheroids were harvested using a pipette and placed into an Eppendorf tube. The medium was removed and spheroids were transferred into a bovine collagen type I (acid extracted, Curacyte Discovery GmbH) gel containing 1 M NaOH, 175 mM NaHCO_3_, 3% FCS, 2 mM L-glutamine, 100 U/mL penicillin, and 100 µg/mL streptomycin. After polymerization for one hour at 37 °C, collagen gels were covered with DMEM. For quantification of spheroid sizes, each spheroid’s radius was measured with the ImageJ software (http://rsb.info.nih.gov/ij; version ImageJ 1.53a). Afterward, the volume was calculated with the formula: $V=\frac{4}{3}\pi r^{3}$.

### 2.3. Tumor Grafting Experiments

Mice with fibroblast-specific deletion of MMP14 were generated by crossing a mouse line with floxed MMP14 gene to mice that express the estrogen receptor inducible Cre recombinase under control a fibroblast-specific regulatory fragment of the pro-α2 (I) collagen gene, as previously described [11]. For experimental skin fibrosis, we used an established mouse protocol [21]. Control mice were treated with intradermal injections of bleomycin (100 µg) (PZN-02411351, Medac GmbH, Wedel, Germany) five days a week for five weeks. Afterward, 0.5 × 10^6^ B16F1 cells were injected intradermally into the mice’s flank and tumor growth was followed and documented. Tumor size was measured using a precision caliper (Mitutoyo, Neuss, Germany) and mice were euthanized when they reached a maximal allowed tumor size. Tumors and organs were collected for further analysis.

### 2.4. Atomic Force Microscopy (AFM)

AFM measurements of peritumoral and dermal tissue were performed on freshly cut 20 µm cryosections using a JPK NanoWizard4 atomic force microscope mounted on a Zeiss Axio observer Z1 widefield fluorescence microscope and operated via JPK SPMControl Software v.6. Cryosections were equilibrated in PBS supplemented with protease inhibitors and measurements were performed within 20 min after sectioning the samples. For micromechanical measurements, spherical silicon dioxide beads with a diameter of 3.5 μm glued onto tipless silicon nitride cantilevers (CP-PNPLSiO-B-5, NanoAndMore GmbH, Wetzlar, Germany) with a nominal spring constant of 0.08 Nm^−1^ were used. Measurements were performed using the quantitative imaging (QI) mode with a pixel time of 500 ms (approach and retraction), ensuring detection of elastic properties only. Forces of up to 2 nN were applied. For each biological replicate, we analyzed 50–200 force curves of various dermal and peritumoral locations. All analyses were performed with JPK Data Processing Software (Bruker Nano GmbH, Herzogenrath, Germany). Before fitting the Hertz model corrected by the tip geometry to obtain the Young’s modulus (Poisson’s ratio of 0.5), the offset was removed from the baseline, the contact point was identified, and cantilever bending was subtracted from all force curves.

### 2.5. Immunoblot

Cells were lysed with RIPA buffer (150 mM NaCl, 50 mM Tris-HCl, 1% NP-40, 0.1% SDS, 0.5% Na-deoxycholate, 5 mM EDTA, 1% Triton X-100, pH8, containing protease inhibitor (PI)) and homogenized using a Mixer Mill instrument (Retsch, Haan, Germany, MM300) at 30 Hz for 2 min. After incubation on ice for 30 min, homogenates were centrifuged at 16,000× *g* for 10 min and extracted proteins were stored at −20 °C. The protein concentration was determined using a Pierce^®^ BCA protein assay (Thermo Fisher Scientific, #23227, Darmstadt, Germany). Here, 10 µg of protein was resolved on a 10% SDS-PAGE and transferred to a nitrocellulose membrane (10600003, AmershamTM ProtranTM, Ø 0.45 µm, GE Healthcare, distributed by Merck, Darmstadt, Germany). Transfer efficiency was confirmed by Ponceau S staining (P7170, Sigma-Aldrich, Schnelldorf, Germany) of the membrane. The membrane was blocked for 1 h at room temperature (RT) in PBS (1X) with 0.05% Tween20^®^ (PBST) containing 5% milk powder and incubated with the primary antibody overnight at 4 °C (mouse α-PCNA, 1:1000, Life Technologies, #133900, distributed by Thermo Fisher Scientific, Darmstadt, Germany; mouse α-actin, 1:2000, MPBiomedicals, #2928H, Eschwege, Germany). After washing three times for 5 min at RT in PBST, the membrane was incubated for one hour at RT with the HRP-conjugated secondary antibody (rabbit α-mouse-HRP 1:2000, P0260, Agilent, Waldbronn, Germany). After washing three times for 5 min in PBST, bound antibodies were visualized using enhanced chemoluminescence (PierceTM ECL Western Blotting Substrate, 32106, Thermo Fisher Scientific, Darmstadt, Germany).

### 2.6. Immunofluorescence Staining

Cryosections were fixed with 1% PFA for 8 min at RT and washed with PBS. Afterward, sections were permeabilized with ice cold acetone (−20 °C) for 5 min, dried, and blocked with 10% NGS in PBS for 30 min at RT. Primary antibody diluted in 1% BSA in PBS was added to the sections and incubated in a humidified chamber overnight at 4 °C (rat α-Ki67 (M7249, Agilent, Waldbronn, Germany); rabbit α-cleaved caspase 3 (9661-S, Cell Signaling, Frankfurt, Germany); rat α-CD31 (557355, BD Biosciences, Heidelberg, Germany); rabbit α-LYVE-1 (ab14917, Abcam, Cambridge, UK); rat α-CD68 (MCA1957GA, BioRad, Feldkirchen, Germany); rat α-CD16/32 (553141, BD Biosciences, Heidelberg, Germany); rabbit α-S100A4 (ab27957, Abcam, Cambridge, UK); goat anti-TRP2 (sc-10451, Santa Cruz, Heidelberg, Germany)). After washing three times with PBS, sections were incubated in α-SMA Cy3 (C6198, Sigma-Aldrich, Schnelldorf, Germany) and α-Ly6G antibodies (127625, Biolegends, Koblenz; Germany), or with secondary antibody in 1% BSA in PBS in a humidified chamber for 1 h at RT (donkey α-goat 488 nm, A11055; goat α-rabbit 594 nm, A11037; goat α-rat 488 nm, A11006; goat α-rat 594 nm, A11007, Invitrogen, distributed by Thermo Fisher Scientific, Darmstadt, Germany)). After washing three times with PBS, sections were mounted with Immumount (Thermo Fisher Scientific, Darmstadt, Germany) and stored at 4 °C. We used ImageJ software to quantify fluorescence intensities and the number of positive cells (http://rsb.info.nih.gov/ij; version ImageJ 1.53a). Ki67-positive cells were counted in sections from the center of different tumor sections and calculated as the percentage of the total numbers of cells (DAPI positive nuclei).

### 2.7. Picrosirius Red Staining

Sections were deparaffinized, stained in Weigert’s iron hematoxylin (Waldeck GmbH, Münster, Germany) for 5 min, and rinsed several times with fresh and lukewarm water. Sections were differentiated in 1% HCl in 70% ethanol until the cytoplasm was destained and rinsed several times in warm water. Tissue sections were stained in 0.5% picrosirius red staining solution (Sirius Red F3B, Merck, Darmstadt, Germany) in saturated picric acid) for 1 h and rinsed twice with 0.5% acetic acid. Then, sections were dehydrated three times with isopropanol, dipped twice in xylol, and embedded in a xylene-based GLC mounting medium (468253, Sakura, Alphen aan den Rijn, Netherlands).

### 2.8. Invasion Assays Using De-Epidermized Devitalized Skin (DDS)

Invasion assay was performed as previously described [22]. Briefly, back skin punch biopsies (8 mm) were incubated in 5 mg/mL Dispase II (#04942078001, Roche Life Science, Penzberg, Germany) in serum-free DMEM overnight (o/n) at 4 °C to remove the epidermis. Biopsies were washed with PBS and devitalized by three cycles of freezing at −80 °C for 30 min and thawing in the water bath at 37 °C. After placing on a metal net, 1 × 10^5^ B16F1 cells were seeded on the dermis and incubated for three weeks at 37 °C and 5% CO_2_ at air–liquid interfaces.

Invasion of cells into the dermis was evaluated by quantifying the migrated distance of single visible melanoma cells and areas of tumor nests (island of clustered melanoma cells) within the dermis (carefully excluding visible hair follicles) on H&E images using ImageJ software.

### 2.9. Second Harmonic Generation

SHG analysis was performed on deparaffinized tissue sections using an upright multiphoton microscope (TCS SP8 MP-OPO, Leica, Wetzlar, Germany) equipped with a Ti:Sa laser (Chameleon Vision II; Coherent, Santa Clara, CA, USA) tuned to 1050 nm. For the acquisition of the pictures, LAS X software (Leica Microsystems) was used. Collagen fiber alignment and waviness were quantified using ImageJ software, as previously described [23,24].

### 2.10. RNA Isolation and Real-Time PCR

For isolation of tissue RNA, we used the Qiagen Fibrous Kit (74704, Hilden, Germany) according to the instructions. RNA was reverse transcribed by adding 1 µg RNA to the master mix (containing reverse transcriptase, RNase inhibitors, oligo-dT primers, dNTPs, and PCR-buffer) and setting thermal cycling conditions to 21 °C for 10 min, then 42 °C for 30 min and 99 °C for 5 min. The cDNA was used for real-time PCR using the StepOne Real-Time kit (Applied Biosystems, distributed by Thermo Fisher Scientific, Darmstadt, Germany). Amplification was performed in a total volume of 20 µL for 40 cycles. Thermal cycling conditions were set to 50 °C for 2 min, 95 °C for 10 min, with 40 cycles of amplification at 95 °C for 15 s and at 60 °C for 1 min for each cycle. S26 was used as a control. Primers for amplifying F4/80, TNF-α, and Fizz have been previously described (Chenery et al., 2019, Ishikawa et al., 2020, Zhou et al., 2018). Primers for the amplification of LOX, LH2, CD68, iNOS, Arginase 1, αSMA, and S26 are listed in Table S1.

### 2.11. Statistics

Statistical analysis was performed using Graphpad Prism software (GraphPad, San Diego, CA, USA). The student’s t-test was used for data analysis, with *p* < 0.05 considered statistically significant. Kolmogorov–Smirnov test was used for AFM analysis.

### 2.12. BrdU (5-bromo-2′-deoxyuridine) Incorporation Assay

B16F1 cells were grown in serum-free DMEM for 24 h before plating in pre-coated 96-well plates for a further 24 h. Cellular proliferation was analyzed using the Cell Proliferation ELISA^®^ Kit (Cell Proliferation ELISA, BrdU kit, Cat. No. 11 647 229 001, Roche Life Science, Penzberg, Germany) according to the manufacturer’s instructions. Using this kit, the pyrimidine analog BrdU was incorporated into the newly synthesized DNA of proliferating cells replacing [^3^H]-thymidine, and then recognized by an antibody conjugate. Cell proliferation is shown as average amount of incorporated BrdU into the DNA of proliferative cells measured at 450 nm wavelength.

## 3. Results

### 3.1. Reduced Melanoma Growth in MMP14^Sf−/−^ Mice

**Figure 1:**
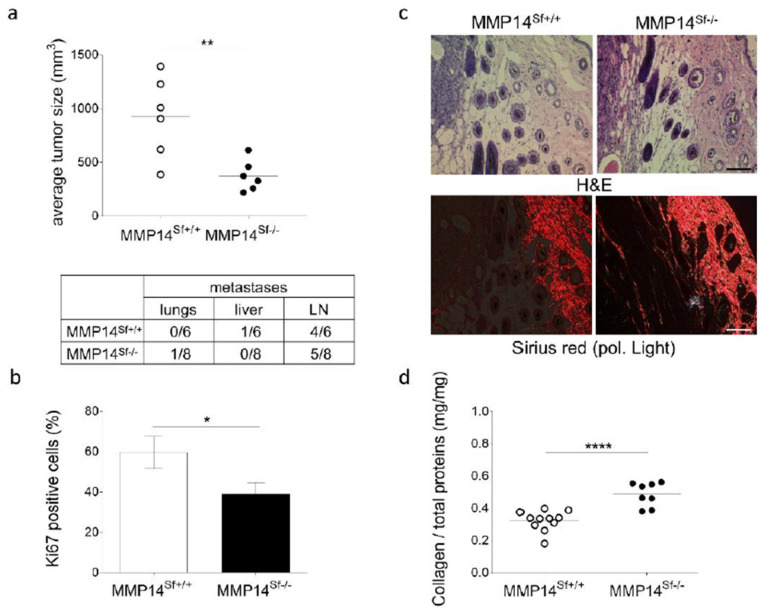
B16F1 melanoma growth in MMP14^Sf+/+^ and MMP14^Sf−/−^ mice. (**a**) Average tumor sizes at day 14 (upper) (MMP14^Sf+/+^ *n* = 6; MMP14^Sf−/−^ *n* = 6) and ratio of organs with metastasis (lower). Shown in the table are the numbers of positive organ metastases. (**b**) Percent of Ki67 positive melanoma cells in the tumor, quantified by immunofluorescence staining. (**c**) Hematoxylin and eosin (H&E) staining (upper) and picrosirius red staining (lower) of grafted B16F1 melanoma, representative pictures. (**d**) Collagen content in peritumoral areas was analyzed by hydroxyproline quantification. Mean +/− SEM; MMP14^Sf+/+^ *n* = 6; MMP14^Sf−/−^ *n* = 8 (* *p* < 0.05, ** *p* < 0.01, **** *p* < 0.0001); scale: 100 µm.

To explore the functional consequence of stromal MMP14 and dysregulated ECM composition in melanoma growth, we used this mouse strain as an in vivo model for melanoma growth. We injected melanoma cells intradermally into the flank of control and MMP14^Sf−/−^ mice and followed the tumor growth over time. Tumors formed in MMP14^Sf−/−^ mice were significantly smaller than in control animals (Figure 1a). This finding was paralleled by a decreased ratio of the proliferation marker (Ki67-positive cells) within the tumor, as detected by immunofluorescence on the tissue (Figure 1b). Despite the differences in tumor growth in MMP14^Sf−/−^ mice, the metastases to lymph nodes, lungs, and liver were not altered (Figure 1a). In the peritumoral tissue of MMP14^Sf−/−^ mice, blood vascularization was reduced compared to control littermates (Figure S1). Lymph vascularization was also reduced, although not significantly. However, most likely this effect was an indirect effect of MMP14 deletion, as in several tumors (2–3) of comparable size, the number of vessels was similar in both genotypes. Deleting fibroblast MMP14 did not alter the number of inflammatory cells detected by immunofluorescence on the tissue (Figure S2a). Among those, macrophages were not changed by tissue immunodetection (CD68) or by transcript analysis (CD68 and F4/80) (Figures S2a and S3). Furthermore, M1/M2 macrophage subtypes were equally detected in peritumoral areas, as observed by amplification of markers for the M1 (iNOS, TNF-α) and M2 (Arginase 1, Fizz) phenotypes. Additionally, detection of markers for the cancer-associated fibroblasts, S100A4/Fsp-1, and α-SMA indicated that ablation of MMP14 in fibroblasts does not reduce the amounts of these cells around tumors (Figure S2).

### 3.2. Alterations in the Peritumoral Matrix of MMP14^Sf−/−^ Mice

Due to impaired collagenolysis, collagen type I was increased in the skin of the MMP14^Sf−/−^ mice [11]. Increased collagen was also found in peritumoral areas of the MMP14^Sf−/−^ mice by histological analysis of fibrillar collagen by picrosirius red staining–polarized light visualization (Figure 1c) and quantification of the hydroxyproline content (Figure 1d). It was shown that independent of the collagen amount, the collagen structure can affect cancer cells and tumor progression by building aligned fibers perpendicular to the tumor [25,26]. The collagen fiber structure and arrangement, as analyzed by second harmonic generation analysis (SHG), showed that the fibers’ alignment (although mainly parallel to the tumor) and waviness were increased comparably in the peritumoral tissue compared to healthy skin in both mice independently of their genotypes (Figure S4).

**Figure 2:**
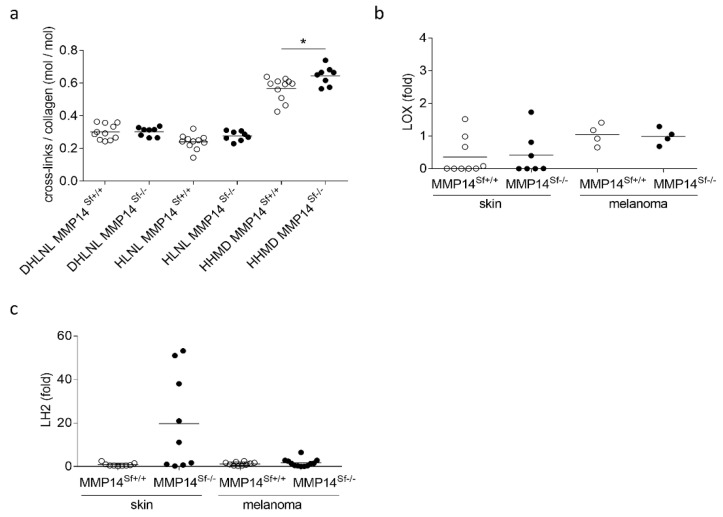
Analysis of skin and peritumoral tissue samples of MMP14^Sf+/+^ and MMP14^Sf−/−^ mice. (**a**) Analysis of collagen cross-linking normalized to collagen content. (**b**) LOX and (**c**) LH2 transcript levels were quantified by quantitative real-time PCR. Note: * *p* < 0.05, mean +/− SD. Each dot represents one specimen per mouse.

In tumors and fibrosis, increased collagen levels and altered collagen cross-linking contribute to enhancing tissue stiffness [27,28]. We analyzed cross-links in the peritumoral areas of age- and gender-matched MMP14^Sf−/−^ and MMP14^Sf+/+^ mice. We did not detect any increase in DHLNL cross-links typical for hard tissues [27]. The cross-links of HLNL and HHMD, which are predominant in soft tissues, were unaltered or showed a slight increase (HHMD) (Figure 2a). The activity levels of the two enzymes, LOX and PLOD2/LH2, which are responsible for the generation and composition of cross-links [29,30], were not altered in MMP14^Sf−/−^ and MMP14^Sf+/+^ mouse tumors (Figure 2b,c). As shown earlier, although we did not observe an alteration of the pattern of cross-links in the skin, we unexpectedly detected increased transcript expression of LH2; the reason and consequences for this are unclear to us. However, we saw increased stiffness in MMP14^Sf−/−^ compared to control skin when we measured tissue tension using a tensile test until tissue failure [11]. We also confirmed this in the skin at the microscopical level using AFM measurements (Figure S5a). A similar increase was detected in peritumoral areas six days post-melanoma cell injection, when tumors in MMP14^Sf−/−^ mice were significantly smaller than in control animals (Figure S5b,c), but not peritumoral tissue at day 13 post-injection. At this time point, stiffness was comparable in both mice genotypes (Figure S5a).

### 3.3. Reduced Area of Melanoma Tumor Nests in Skin Composites

**Figure 3:**
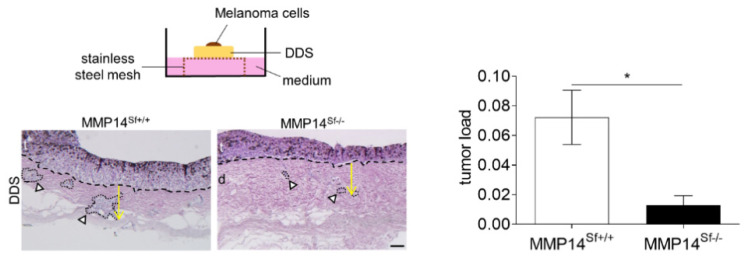
In vitro DDS (de-epidermized devitalized skin) invasion assay. In the upper inset image, the DDS assay scheme is shown. Below, H&E staining of DDS skin sections after three weeks of the invasion is shown. The border between melanoma cells and the dermis is marked by the dashed black line and invaded tumor nests (white arrowhead) in the dermis are marked by a dotted line. The graph underneath depicts the sizes of the total tumor nests in µm^2^ per µm^2^ skin. Mean +/− SEM; MMP14^Sf+/+^ *n* = 3; MMP14^Sf−/−^ *n* = 3; * *p* < 0.05; t = tumor; d = dermis; scale: 100 µm.

Although we showed that numbers of CAFs and inflammatory cells (Figure S2) were not altered, their function can be modified and they can contribute differently to melanoma growth [31,32]. To address if changes of the extracellular composition induced by deletion of fibroblast MMP14 are sufficient to affect melanoma growth, we used an ex vivo invasion system [22]. In this model, murine B16F1 melanoma cells were seeded on the papillary dermis of de-epidermized devitalized skin (DDS) samples collected from MMP14^Sf−/−^ or control animals, and tumor invasion was followed over time (Figure 3). The sizes of tumor nests grown within the dermis of MMP14^Sf−/−^ mice were significantly reduced. However, the invasion depth remained unchanged (Figure 3). These data suggest that ECM changes caused by the lack of MMP14 production in fibroblasts are sufficient to inhibit melanoma growth.

### 3.4. High Collagen Type I and Tissue Tension Levels Negatively Modulate Melanoma Growth

In vitro, both collagen levels and stiffness were associated with reduced or enhanced melanoma proliferation and invasion [33,34,35,36]. Our in vivo data showed that deletion of MMP14 in fibroblasts is associated with increased collagen type I, skin stiffness, and melanoma growth inhibition levels (Figure 1 and Figure S4).

**Figure 4:**
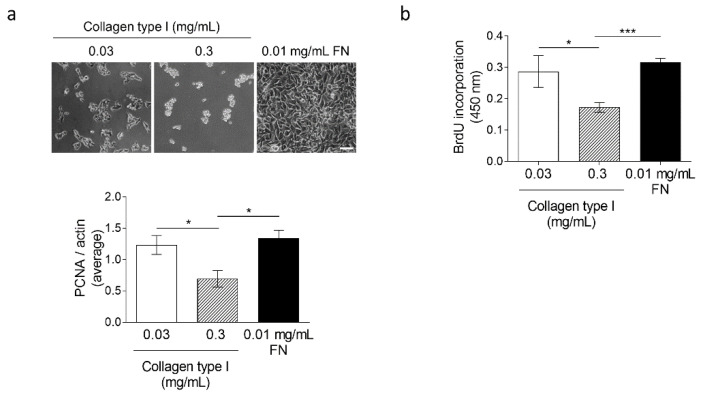
Analysis of B16F1 cell proliferation on collagen type I-coated stiffness plates. B16F1 melanoma cells were cultured for 48 h on culture plates coated with different concentrations of collagen type I and fibronectin as control. (**a**) Phase contrast images of cultures and average densitometry values of PCNA (Proliferating cell nuclear antigen) in immunoblot normalized to actin in three independently performed experiments were averaged and are expressed in the graph. (**b**) BrdU (5-bromo-2′-deoxyuridine) incorporation measurements in the same culture conditions. Mean +/− SEM. In the graph, the average size of *n* = 5–8 biological replicates of each condition is shown. Note: * *p* < 0.05; *** *p* < 0.001; scale: 100 µm.

To address whether increased collagen or tension inhibits melanoma growth, we used an in vitro approach. We seeded melanoma cells on stiff surfaces (1GPa) coated with either low (0.03 mg/mL) or high (0.3 mg/mL) concentrations of collagen type I. Fibronectin (0.01 mg/mL) was used as a positive control. After 48 h, melanoma cell proliferation was analyzed by quantitating the levels of PCNA (Figure 4a) and through BrdU incorporation (Figure 4b). At low collagen concentrations, PCNA levels and incorporation of BrdU were comparable to the fibronectin control but decreased at high concentrations (Figure 4a,b). These results indicate that high amounts of collagen type I negatively modulate melanoma growth.

**Figure 5:**
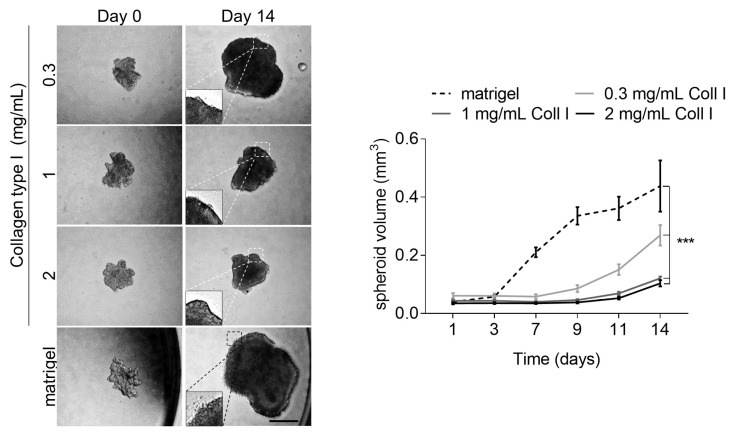
B16F1 spheroid growth in fibrillar collagen type I gels and Matrigel. Mean +/− SEM. In the graph, the average size of *n* = 5–9 biological replicates of each condition is shown. Note: *** *p* < 0.001; scale: 500 µm.

To ascertain whether the environment’s structure would differently affect melanoma cell proliferation, we cultured melanoma cells as spheroids in a three-dimensional fibrillar collagen gel. We used increasing collagen type I concentrations (0.3, 1, and 2 mg/mL) and Matrigel as a positive control (Figure 5). On day three, the average spheroid size in Matrigel, but not in collagen, started to increase steadily. In contrast, the growth of melanoma spheroids embedded at the lowest collagen concentration (0.3 mg/mL) increased on day 9. After day eleven, we detected a modest increase in growth in 1 and 2 mg/mL collagen type I gels, which resulted in significantly smaller spheroids than in Matrigel or low collagen cultures on day 14 (Figure 5). Interestingly, only cells from spheroids grown in Matrigel invaded into the surrounding matrix (Figure 5, inserts).

### 3.5. Increased Tissue Stiffness and Matrix Density Levels Lead to Reduced Melanoma Growth In Vivo

**Figure 6:**
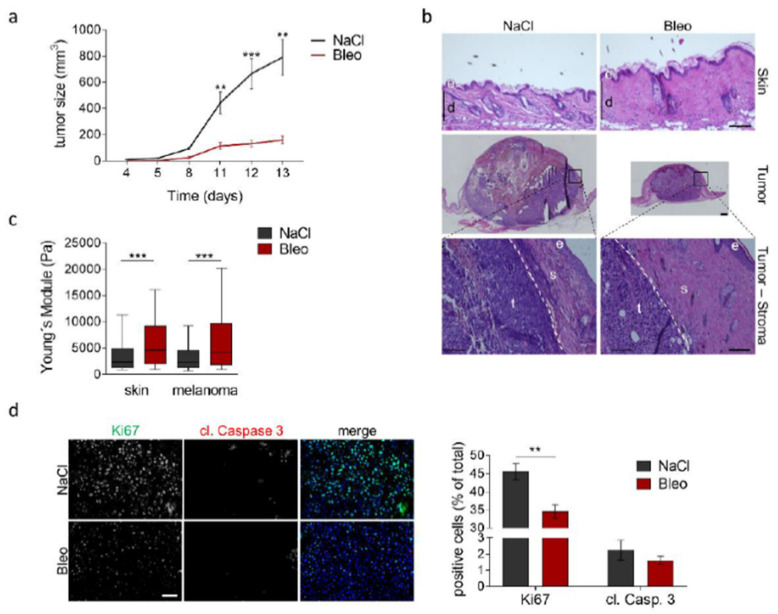
Melanoma growth in a fibrotic environment. (**a**) B16F1 melanoma growth in mice treated with bleomycin (Bleo) induced a fibrotic skin lesion or NaCl as control. (**b**) H&E staining of representative skin and tumors of bleomycin- and NaCl treated mice. Magnification of tumor-stroma areas shows the persistence of peritumoral fibrosis in bleomycin-treated mice. (**c**) AFM measurement of peritumoral tissue (melanoma) and skin (skin) after NaCl control and bleomycin treatment. NaCl *n* = 8; Bleo *n* = 7; ** *p* < 0.01; *** *p* < 0.001; e:epidermis; d:dermis; s:stroma; t:tumor; scale:100 µm. (**d**) Immunofluorescence staining for Ki67 and cleaved (cl.) Caspase 3 in melanoma grown in NaCl- and bleomycin-treated mice and quantified positive cells (lower graph). Mean +/− SEM; NaCl *n* = 8; Bleo *n* = 7; ** *p* < 0.01; scale: 50 µm.

To confirm that increases in collagen and tissue stiffness levels affect melanoma development in vivo, independently of MMP14 deletion in fibroblasts, we undertook a different in vivo approach. We induced in wild-type mice the formation of fibrotic, collagen-rich skin areas by daily intradermal injections of bleomycin or NaCl as control over four weeks. Melanoma cells were injected intradermally into the fibrotic skin and tumor growth was followed over time (Figure 6). We found melanoma growth to be markedly decreased in fibrotic skin (Figure 6a,b). Consistently, AFM measurements verified increased matrix stiffness of treated peritumoral areas, as well as the more distal dermis (Figure 6c). Analysis of Ki67 and cleaved caspase 3 showed a significant reduction in melanoma proliferation in fibrotic skin but only minor alterations in apoptosis (Figure 6d). These data further underscore that a matrix-rich and stiff microenvironment negatively influences melanoma growth.

## 4. Discussion

The homeostatic balance between ECM synthesis and degradation is altered in many skin diseases, including cancer. Several proteases contribute to matrix breakdown, including the matrix metalloproteinases (MMPs) produced by either tumor or stromal cells [37]. MMP14 is expressed in melanomas at the early time point of disease development, and later its expression correlates with disease progression and patient outcome [38]. While several studies have demonstrated how MMP14 expression in melanoma cells can contribute to the pathogenesis of melanomas [12], it is unclear how MMP14 expression in fibroblasts contributes to melanoma progression. In the skin, by deleting MMP14 in fibroblasts, we previously showed that lack of this particular crucial collagenase leads to the generation of the ECM-rich stiff dermis without altered inflammation or tissue vascularization [11]. Our MMP14^Sf−/−^ mice model provided us with the great opportunity to directly ask whether loss of MMP14 and the arising resulting collagen-rich stiff ECM promotes or inhibits the growth of melanomas in vivo. Melanomas grafted in this environment showed lower growth rates than controls and did not display altered infiltration of inflammatory cells. Using in vitro invasion systems devoid of cellular activities, we determined that the structural peritumoral environment generated in the absence of MMP14 in fibroblasts has an inhibitory effect on the growth of melanomas.

We detected increased stiffness in the skin and the peritumoral areas of early but not late MMP14^Sf−/−^ mice tumors compared to control mice. It is possible that after tumor growth increased and many more cells entered the tumor microenvironment, tumors partly overcame the restraints of the tissue by loosening the ECM and dissipating stiffness around the tumors. Such a function was shown to be controlled by the contractile and remodeling activity of cancer-associated fibroblasts [32,39]. Using markers for CAFs, we could not detect an increased number of these cells at the later time point as compared to controls. Possibly, to sustain the stiffness in peritumoral areas over time, a relative increase in these cells is necessary. As demonstrated for various tumors, an alternative is that internal forces from the growing tumor pushing toward the surrounding stroma allow the tumor to expand [40]. The expanding melanoma cells that express several proteases can release tissue tension via localized proteolysis [7,41,42].

During cancer progression, especially in many epithelial types, increased peritumoral collagen accumulation and enhanced cross-linking lead to stiffened tissue [26,43]. In skin [11] and peritumoral areas of MMP14^Sf−/−^ mice, we detected increased collagen content, however the amounts and pattern of collagen cross-links were not altered compared to controls. Comparably, the two enzymes mediating this process at different levels, LH2 and LOX, were not changed. Although we did not detect alterations in the collagen cross-links in the MMP14^Sf−/−^ mice, increased matrix stiffness may result from increased collagen or collagen cross-linking through non-enzymatic glycation [44]. These types of cross-links increase during aging [44]. As the mice we used for the experiments were young, we excluded glycation as a major cause of cross-linkage, however a possible effect of these links cannot be entirely excluded. Previous studies showed that increased matrix density results in decreased angiogenesis. In contrast, increased matrix stiffness independent of matrix density induces increased angiogenic outgrowth, invasion, and neovessel branching [45]. Along these lines, reduced angiogenesis in MMP14^Sf−/−^ mice may also be due to increased matrix density in peritumoral areas. These data suggest that the effects of matrix density and cross-linkage can have effects independent of each other.

In agreement with our previous data, the increased collagen density in the skin of MMP14^Sf−/−^ mice could contributed to altering the mechanical properties of the ECM independently of enhanced cross-linkage. These structural alterations may also hinder cell migration [46,47]. However, in the DDS assay analysis, we detected a similar migratory capacity of single invading melanoma cells in the MMP14^Sf−/−^ and control mice dermis samples. This fact was not surprising, as melanoma cells can express MMP14 and navigate small paths generated in the ECM.

On the contrary, the expansion of tumor cells from the nests was impaired. These data would suggest that the growth of melanoma cells and the development of tumor nodules formed within the matrix need support from an active stroma providing stimuli or larger paths favoring outgrowth in the surrounding matrices. In support of these possibilities, it was shown that the cross-talk with the tissue stroma generates tracks of proteins that support collective migration through heterologous cell–cell contacts with stromal fibroblasts [48]. Furthermore, fibroblasts continually regulate the ECM changes in healthy tissue by strictly controlling MMP1, MMP2, MMP9, MMP13, and MMP14 activities [49]. Expression of MMP14 in fibroblasts can regulate activation of MMP2 or MMP13 in peritumoral stromal areas [50,51], and deletion of MMP14 in fibroblasts results in a significant reduction of MMP2 activation [11]. A lack of MMP14 expression in fibroblasts would dramatically reduce proteolysis in peritumoral areas and affect melanoma outgrowth. Thus, the stromal fibroblast activation of these proteases may support the idea of secondary widening being necessary during the collective invasion.

We have shown that increased collagen density and tissue tension resulting from MMP14 deletion in fibroblasts leads to melanoma growth inhibition. However, it is unclear whether this is the direct consequence of deletion of MMP14 or whether an environment with high collagen, matrix density, and altered mechanics levels would be sufficient to inhibit melanoma growth. To date, although collagen and tissue rigidity are documented players in cancer progression, the role of these factors in the melanoma environment is quite controversial [33,34,36]. However, when we implanted melanoma cells into fibrotic lesions generated by bleomycin injections [21], melanoma growth in the stiff, collagen-rich tissue was significantly reduced. This result supports inhibitory roles of stiffness and collagen density in melanoma cell proliferation. Additionally, melanoma cell proliferation in vitro was reduced when cells grew in high concentrations of collagen type I on a two-dimensional stiff substrate (1GPa) or as spheroids in three-dimensional collagen gels.

The negative effects of increased collagen density in melanoma growth in mice is supported by different observations made in patients. For example, human desmoplastic melanoma is characterized by an abundant fibrous matrix and a more favorable prognosis than nodular melanoma with low matrix accumulation [52]. Furthermore, a fibrotic dermis is also found in late regressing human melanoma, possibly suggesting an anti-tumor effect of collagen [53]. Another study analyzing various grades of regressing melanoma showed a weak association of inflammation in late and intermediate regressive melanomas, but fibrosis dominated the histologic picture [54]. Whether or not fibrosis directly contributed to melanoma regression was not addressed, however based on our findings, we could hypothesize it does. Of further note, in patients with various forms of fibrosis, there is an increased risk for non-melanoma types of skin tumors [55], likely owing to increased inflammation, however not of melanoma-type tumors. These observations and our data suggest that increased collagen accumulation is an obstacle to melanoma growth.

## 5. Conclusions

Altogether, our data demonstrate that loss of MMP14 activity in fibroblasts leads to enhanced tissue density and tension, thus inhibiting melanoma growth by attenuating angiogenesis and tumor cell proliferation. Furthermore, these data highlight the negative roles of collagen and stiffness in melanoma growth.

## Data Availability

Data generated or analyzed during this study are included in this article.

## Supplementary Materials

The following are available online at https://www.mdpi.com/article/10.3390/cancers13081984/s1: Figure S1: Immunofluorescence staining for CD31 (green) and LYVE-1 (red) of tumor–stroma tissue. The graphs depict the average positive cells in percentages. Note: ** *p* < 0.01; MMP14^Sf+/+^ *n* = 4–5; MMP14^Sf−/−^ *n* = 7; e: epidermis; s: stroma; t: tumor; scale: 100 µm, Figure S2: (**a**) Immunofluorescence staining for macrophages (macro, CD68, red), neutrophils (neutro, Ly6G, green), and NK cells (CD16/32, green) of tumor–stroma tissue. (**b**) Immunofluorescence staining for S100A4 (red) and the melanoma marker Trp2 (tyrosine-related protein 2, green) of tumor–stroma tissue. The graph depicts S100A4-positive cells. MMP14^Sf+/+^ *n* = 7; MMP14^Sf−/−^ *n* = 5. (**c**) Transcriptional analysis of αSMA in peritumoral tissue. MMP14^Sf+/+^ *n* = 6; MMP14^Sf−/−^ *n* = 8; e: epidermis; s: stroma; t: tumor; scale: 100 µm, Figure S3: Transcriptional analysis of markers for macrophages (CD68 and F4/80) and for M1 (iNOS, TNF-α) and M2 (Arginase1, Fizz) macrophage subtypes in peritumoral tissues. MMP14^Sf+/+^ *n* = 4–10; MMP14^Sf−/−^ *n* = 3–12, Figure S4: Second harmonics generation analysis of skin and peritumoral tissue. Collagen fiber alignment and waviness were determined using coherency (alignment) and straightness (waviness) parameters, with average quantifications shown in the graphs. Peritumoral tissue (melanoma); scale: 100 µm; *n*.s., not significant; * *p* < 0.05; *** *p* < 0.001. MMP14^Sf+/+^ *n*= 8; MMP14^Sf−/−^ *n*= 8, Figure S5: Atomic force microscopy (AFM) of peritumoral tissue (melanoma) and skin. (**a**) Analysis of tissue stiffness, Young’s modulus values in melanomas at 13 days (200–500 tissue measurements per mouse, MMP14^Sf+/+^ *n* = 7; MMP14^Sf−/−^ *n* = 7). (**b**) Average tumor size (MMP14^Sf+/+^ *n* = 8; MMP14^Sf−/−^ *n* = 7) and (**c**) tissue stiffness (50–200 tissue measurements per mouse, MMP14^Sf+/+^ *n* = 2; MMP14^Sf−/−^ *n* = 2) at day 6 after melanoma intradermal injection. Note: * *p* < 0.05; **** *p* < 0.0001, Table S1: Primers used for qPCR amplification.
