# Supplementary material for: Fibroblast MMP14-Dependent Collagen Processing Is Necessary for Melanoma Growth
Source: Cancers (Basel). 2021 Apr 20;13(8):1984. doi: 10.3390/cancers13081984 (PMC8074311; doi:10.3390/cancers13081984)
Supplement: Supplementary file 1 [file cancers-13-01984-s001.zip › cancers-1144215-supplementary.pdf]

Supplementary Materials:

Fibroblast MMP14-Dependent Collagen Processing is Necessary for Melanoma Growth

Elke Pach, Jürgen Brinckmann, Matthias Rübsam, Maike Kümper, Cornelia Mauch and Paola Zigrino

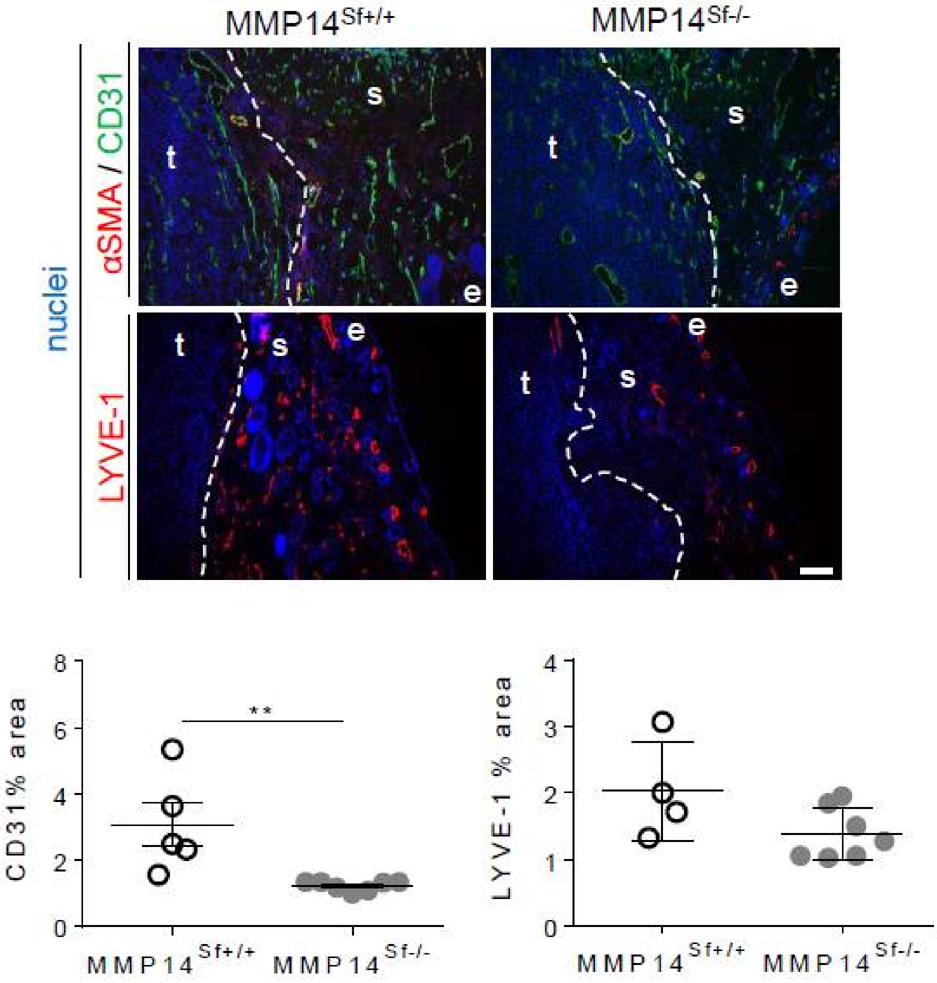

Figure S1. Immunofluorescence staining for CD31 (green) and LYVE-1 (red) of tumor–stroma tissue. The graphs depict the average positive cells in percentages. Note: ** $p < 0.01$; MMP14$^{Sf+/+}$ $n = 4–5$; MMP14$^{Sf-/-}$ $n = 7$; e: epidermis; s: stroma; t: tumor; scale: 100 µm.

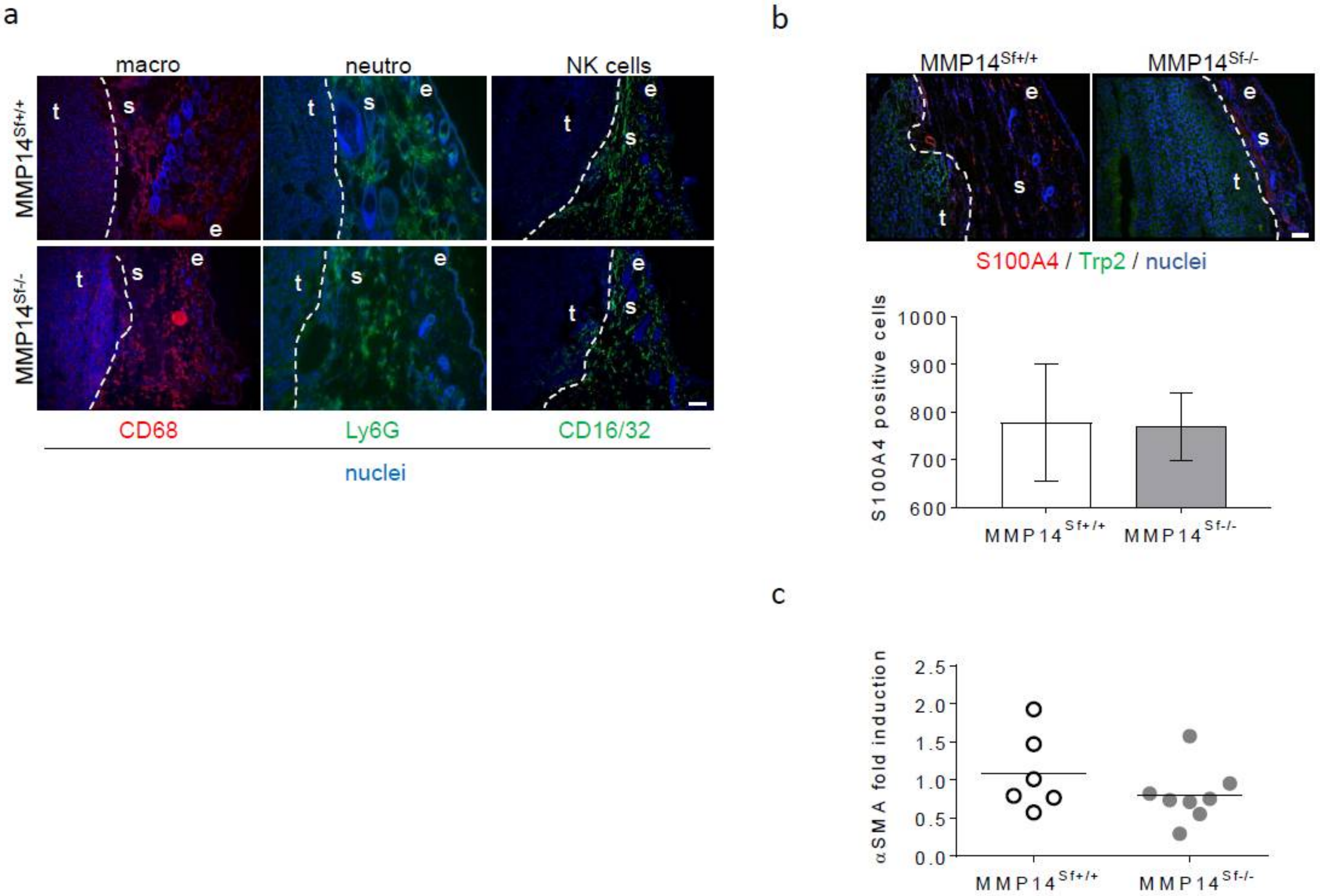

Figure S2. (**a**) Immunofluorescence staining for macrophages (macro, CD68, red), neutrophils (neutro, Ly6G, green), and NK cells (CD16/32, green) of tumor–stroma tissue. (**b**) Immunofluorescence staining for S100A4 (red) and the melanoma marker Trp2 (tyrosine-related protein 2, green) of tumor–stroma tissue. The graph depicts S100A4-positive cells. MMP14$^{Sf+/+}$ $n = 7$; MMP14$^{Sf-/-}$ $n = 5$. (**c**) Transcriptional analysis of αSMA in peritumoral tissue. MMP14$^{Sf+/+}$ $n = 6$; MMP14$^{Sf-/-}$ $n = 8$; e: epidermis; s: stroma; t: tumor; scale: 100 μm.

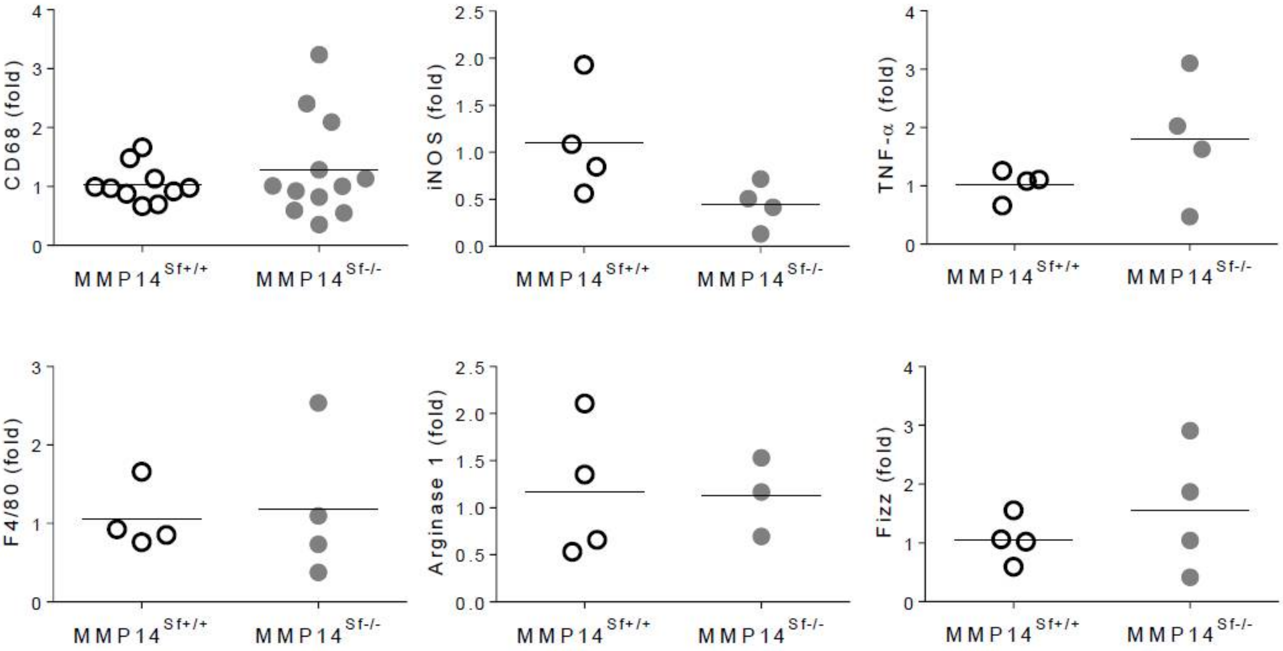

Figure S3. Transcriptional analysis of markers for macrophages (CD68 and F4/80) and for M1 (iNOS, TNF-α) and M2 (Arginase1, Fizz) macrophage subtypes in peritumoral tissues. MMP14$^{Sf+/+}$ n = 4–10; MMP14$^{Sf-/-}$ n = 3–12.

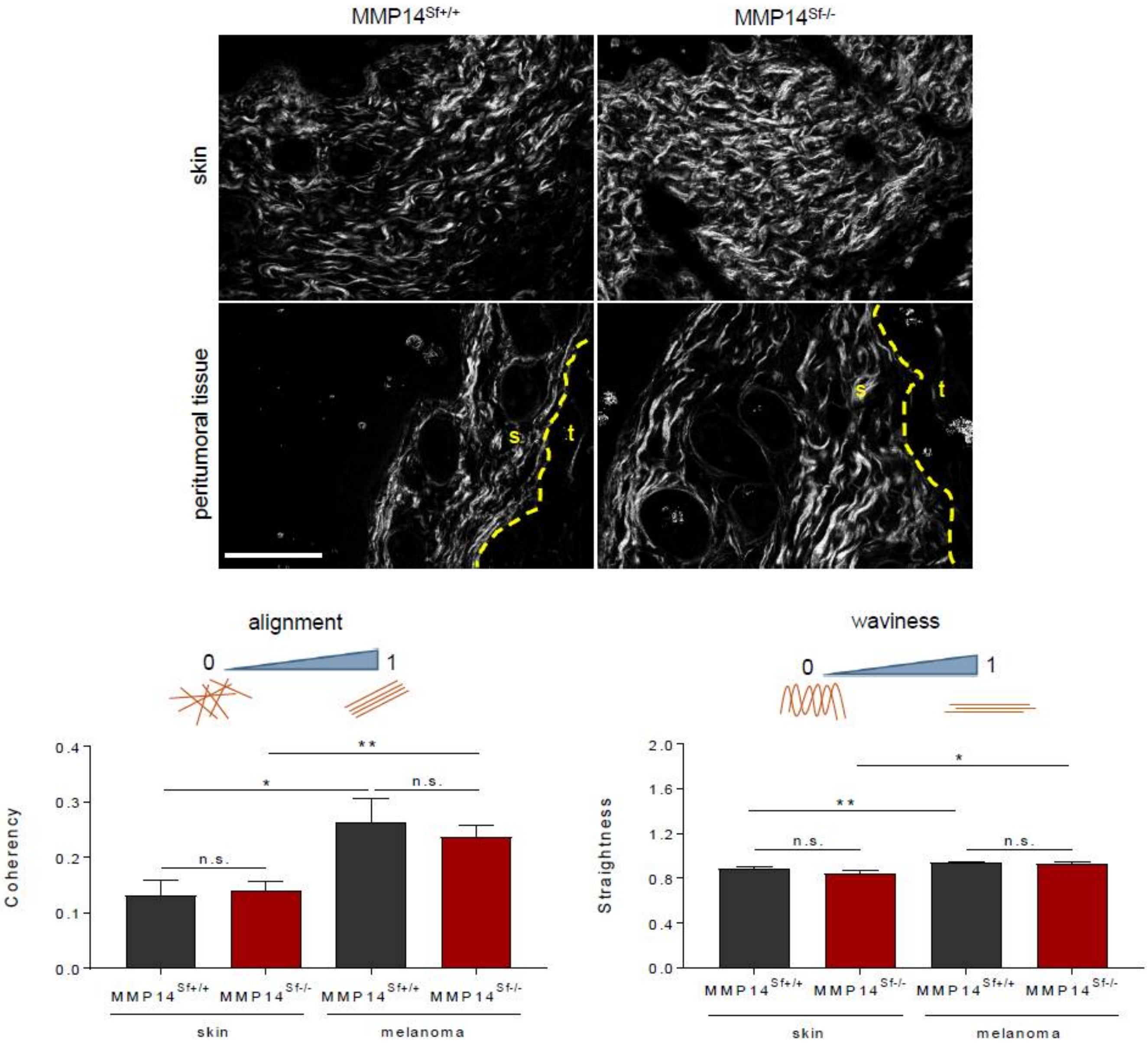

Figure S4. Second harmonics generation analysis of skin and peritumoral tissue. Collagen fiber alignment and waviness were determined using coherency (alignment) and straightness (waviness) parameters, with average quantifications shown in the graphs. Peritumoral tissue (melanoma); scale: 100 µm; *n*.s., not significant; * $p < 0.05$; *** $p < 0.001$. MMP14$^{Sf+/+}$ n= 8; MMP14$^{Sf-/-}$ n=8.

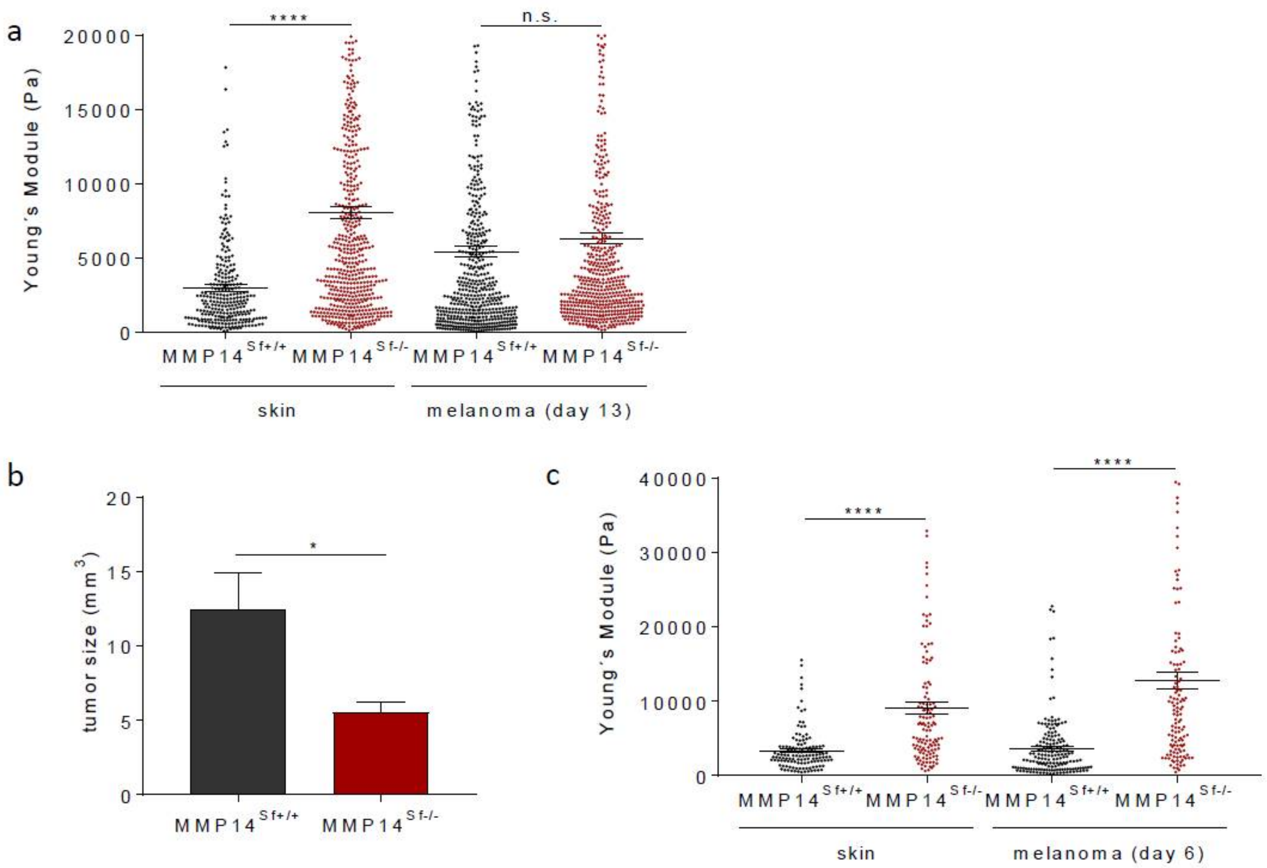

Figure S5. Atomic force microscopy (AFM) of peritumoral tissue (melanoma) and skin. (**a**) Analysis of tissue stiffness, Young's modulus values in melanomas at 13 days (200–500 tissue measurements per mouse, $MMP14^{Sf+/+}$ $n = 7$; $MMP14^{Sf-/-}$ $n = 7$). (**b**) Average tumor size ($MMP14^{Sf+/+}$ $n = 8$; $MMP14^{Sf-/-}$ $n = 7$) and (**c**) tissue stiffness (50–200 tissue measurements per mouse, $MMP14^{Sf+/+}$ $n = 2$; $MMP14^{Sf-/-}$ $n = 2$) at day 6 after melanoma intradermal injection. Note: * $p < 0.05$; **** $p < 0.0001$.

Table S1. Primers used for qPCR amplification.

Gene		Primer seq. (5'-3')	amplified fragment
LOX	for	AGCTACCTGGTGCCTGAATC	128bp
	rev	ACTGGGAACTGGGCTTCTTT	
LH2	for	TTGTATTTGCTGGTGGGCCT	144bp
	rev	GGTAGCGTTTCCCAATGTGC	
CD68	for	AGCTGCCTGACAAGGGACAC	203bp
	rev	CGCTCCTTGGTGGCTTACAC	
iNOS	for	TGCTCCCTTCCGAAGTTTCT	153bp
	rev	ACTCTCTTGCGGACCATCTC	
Arginase 1	for	GCTTCGGAACTCAACGGGAGGG	215bp
	rev	ACCAGAAAGGAACTGCTGGGATACA	
α SMA	for	CAGCGGGCATCCACGAA	218bp
	rev	CCCACCGATCCAGACAGA	
S26	for	AATGTGCAGCCCATTCGCTG	325 bp
	rev	CTTCCGTCCTTACAAAACGG	
